# Critical Care Management of a Severe Acute Respiratory Distress Syndrome COVID-19 Patient With Control Cesarean Section

**DOI:** 10.7759/cureus.22660

**Published:** 2022-02-27

**Authors:** Eduardo E Chang, Marcos Cordoba, Sruthi Vellanki, Anup Kumar Trikannad Ashwini Kumar, Esther Segura

**Affiliations:** 1 Pulmonary and Critical Care Medicine, Union Hospital, Terre Haute, USA; 2 Pulmonology, Sleep and Critical Care, Indiana University School of Medicine, Terre Haute, USA; 3 Maternal Fetal Medicine, Michigan State University College of Human Medicine, Grand Rapids, USA; 4 Internal Medicine, Union Hospital, Terre Haute, USA; 5 Hospital Administration, Methodist Hospital Systems, Houston, USA

**Keywords:** prone ventilation, high-risk pregnancy, covid-19 in pregnancy, acute respiratory distress syndrome [ards], covid 19

## Abstract

We share our experience of one 29-year-old female, G2 P1, with acute respiratory distress syndrome (ARDS) and at 30 weeks of pregnancy. The 30-week gravid uterus in combination with a poor ventilation-perfusion ratio creates a restrictive lung pattern that may prove to be lethal for both the mother and baby. Due to her rapid deterioration and increased hemodynamic instability we opted for controlled delivery in the operating room with an ICU physician, a Neonatologist, and an Obstetric team. At 3.27 minutes from induction, the baby was born with Apgar scores of 7 and 8. The mother was placed on a RotoProne® bed, treated with remdesivir, steroids, and was subsequently extubated seven days later. The newborn was admitted to the Neonatal Intensive Care Unit (NICU) after delivery. We have reviewed the literature and provided a concise set of recommendations based on our field experience and current world literature review. Prompt delivery in a controlled environment with multiple resuscitating teams provided expeditious treatment of both patients, maintaining oxygenation and perfusion while keeping hemodynamic stability. The controlled environment and the proximity of all teams avoided deleterious consequences to the unborn baby. This is an example where the risk of keeping the baby in the womb outweighs the premature delivery into a NICU. Both mother and baby were downgraded from their respective Intensive Care Units (ICUs) and discharged home in one month.

## Introduction

Acute respiratory distress syndrome (ARDS) is a severe presentation of acute hypoxic respiratory failure. The number of pregnant women being admitted with coronavirus disease 2019 (COVID-19) infection has increased. Considering new variations and increased vaccinations among the older generations, we expect to see younger patients in our ICUs. The CDC has proposed that pregnancy carries an increased risk for needing either mechanical ventilation or extracorporeal membrane oxygenation (ECMO); overall, mortality seems to have increased, and more recent data suggested a higher rate of intrauterine fetal demise. In general, the presentation of ARDS during pregnancy is an uncommon occurrence. Prior to this pandemic, ARDS was seen with amniotic fluid embolism, aspiration pneumonia, Mendelson syndrome, influenza pneumonia, blood transfusion, sepsis, and trauma; these were the more common causes of ARDS in pregnancies. Pregnant women have increased mortality rates and higher complications due to viral infections when compared with the overall general population. There is a fine balance in the immune system between protecting both the fetus and the mother from foreign pathogens while also allowing the fetus, which can also be perceived as a foreign object in the mother’s body, to grow. Pregnancy is associated with numerous maternal changes in immune function (immunosuppression) and cardiac physiology. These changes could affect susceptibility to numerous infections including viral respiratory infections like severe acute respiratory syndrome coronavirus 2 (SARS-CoV-2). The maternal immune system has the main purpose is of protecting the unborn baby from foreign infections [1,2]. SARS-CoV-2 has been shown to cause intrauterine growth, premature delivery, and increased perinatal deaths. At the time we present this case over 22,000 pregnant patients with COVID-19 had been found to be hospitalized in the USA [2,3]. Around 13% of gravid patients who get COVID-19 can progress to severe disease status, and approximately 4% of those require ICU admission. Compared to the non-COVID-19 group, women in the COVID-19 group had an increased frequency of admission to ICU, mortality, preeclampsia/eclampsia, gestational hypertension, postpartum hemorrhage, spontaneous delivery, preterm birth, fetal distress, and Cesarean section [4]. The Delta variant (B.1.617.2 ) has been also been reported to have increased morbidity among pregnant women with COVID-19, particularly in areas where vaccination rates have been low. The proportion of these cases is rising, and the severity of variant B.1.617.2 in pregnancy is unclear [5].

Infections by SARS-CoV-2 cause severe acute lung injury due to direct damage to the pneumocytes and acute inflammatory changes. The lungs become stiff, with patients requiring mechanical ventilation assistance, prone ventilation, nitric oxide, antivirals, steroids, and vasopressors. Case reports have also reported on treating ARDS patients with veno-venous (VV) ECMO in pregnant patients with successful delivery [6]. Nitric oxide has been used in some cases of ARDS; it has shown improvement in oxygenation but no change in mortality. Its use has also been reported in pregnant patients with the COVID-19 case series [7]. The positive pressure of ventilation and severe hypoxemia with low arterial oxygen can be deleterious to a baby, making the womb a non-hospitable place for the unborn baby. Furthermore, in order to treat hypoxemia, the use of positive end-expiratory pressure (PEEP) and mechanical ventilation in these patients can also cause increased intrathoracic pressure, leading to decreased cardiac output and hypotension [8,9]. The use of vasoactive agents, such as norepinephrine, vasopressin, and large volumes of (IV) fluids can affect the cardiorespiratory system of the unborn baby.

It is important to realize that most of the therapies adopted during this pandemic have been from data of ARDS patients, which tend to be older patients with chronic diseases. The management of respiratory distress in pregnancy has been done from extrapolation of non-pregnant patients, as the incidence of ARDS in pregnancy prior to the pandemic was very rare. In the third trimester, the pressure of the uterus due to anatomical changes can reduce expiratory reserve volume, inspiratory reserve volume, and functional residual capacity. Thus, increasing the risk of severe hypoxemia in pregnant patients, particularly those who are seriously ill. Information concerning delivery timing and acute respiratory distress syndrome are limited, and it is acceptable to consider an early delivery in the setting of deteriorating or persistent critical illness after 30-32 weeks gestation. In our patient, premature delivery was planned due to continued deterioration despite high flow and noninvasive ventilation, the mother’s arterial oxygen content decreased in the 60% range. The patient became progressively tachypneic, the need for pronation, intubation, high PEEP, and possible low tidal volume with high PEEP strategies were contemplated. Many patients in similar circumstances require paralytic agents, high sedation, and time to improve [9,10]. 

## Case presentation

**Figure 1:**
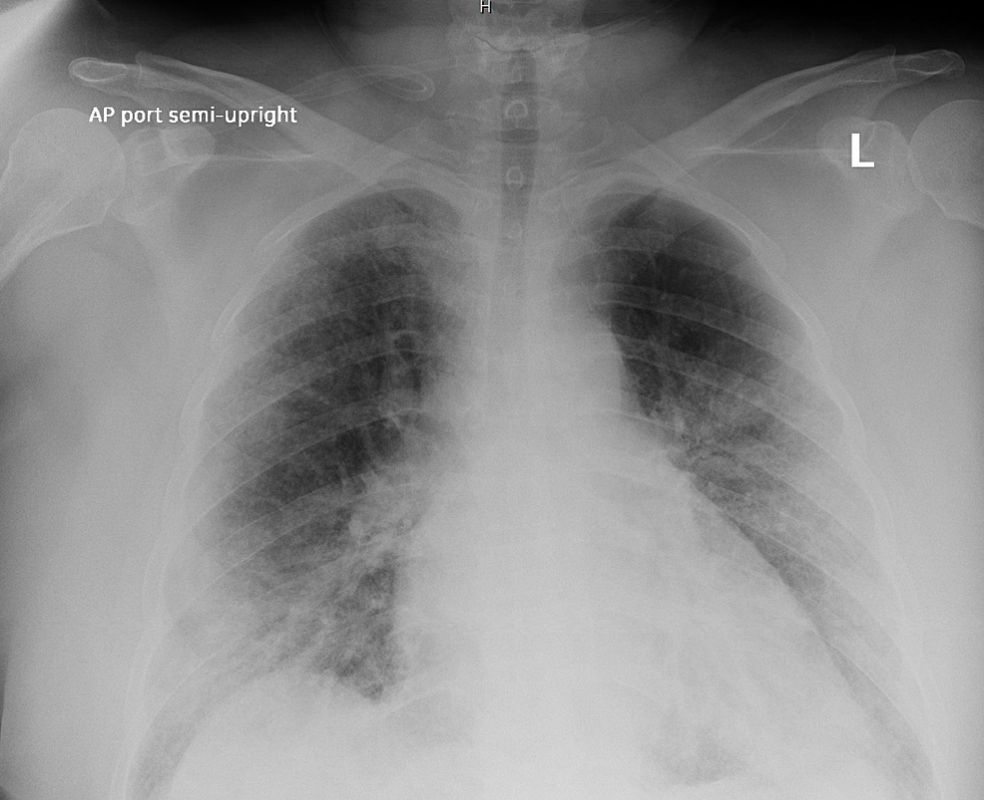
Chest radiograph with bilateral ground-glass opacities, characteristic of COVID-19 pneumonia

A 29-year-old pregnant woman was hospitalized at 30 weeks of gestation. She was a G2 P1, nonsmoker with no significant past medical history. She came to the hospital with an increased cough, fever, wheezing and was admitted to the Obstetric COVID floor. At the time of admission, she required continuous oxygen at 5 liters. On her second day of hospitalization, her oxygenation status deteriorated, and she required a high flow system. She was treated with remdesivir, steroids, high flow oxygen and we decided to transfer her to the ICU. Polymerase chain reaction (PCR) led to the confirmation of COVID-19 viral infection. Chest radiograph revealed bilateral ground-glass opacities, characteristics of COVID-19 pneumonia (Figure 1). Despite the initiation of steroids, remdesivir, and antibiotics, her overall condition continued to decline. Laboratory testing provided: white blood cell count (WBC) was 16 K/$\mu$L (4K/$\mu$L-10K/$\mu$L), beta natriuretic peptide (BNP) of 750 pg/mL (normal low 0 pg/mL - normal high 100 pg/mL), ferritin of 300 ng/mL (normal low 13 ng/mL - normal high 150 ng/mL) and a D dimer > 4 mg/L (normal high 0.5mg/L). The rest of the chemistries and liver function exams were initially normal. The patient continued to be tachycardic, despite further fluid administration and increased oxygen supplementation. The fetal cardiac monitor showed good variability with positive accelerations and no decelerations. viability during this time with no evidence of distress. After careful discussion with the mother and the obstetrician, it was decided to take the patient to the operating room for a controlled Cesarean section. A coordinated and controlled delivery with a team integrated by an intensive care specialist, an obstetrician, and a neonatologist was planned. The prompt delivery was necessary because the patient required intubation and the need for prone ventilation was imminent. The patient was induced by anesthesia, intubated and the Cesarean section was started immediately yielding the delivery of a healthy 8-pound 4-ounce baby boy. The baby’s Apgar scores were 7 and 8, he was taken to the Neonatal Intensive Care Unit by a neonatologist. The ICU team took the mother after surgery to the Medical Intensive Care Unit. Norepinephrine and vasopressin were started after the delivery. The mother was placed on mechanical ventilation with Assist Control mode with a PEEP of 14 and 100% oxygen, being subsequently placed in prone. She was scheduled on 16-hours prone and 8 hours supine. The oxygen partial pressure to fractional inspired oxygen (PaO2/FiO2) ratios and arterial blood gases (ABGS) were checked daily. Over the next 48 hours, she required vasopressin, norepinephrine, steroids, remdesivir, and anticoagulants. The mother was successfully weaned and extubated about one week later. Post extubation, she required high flow oxygen with noninvasive ventilation. On her second week, she was weaned to 5 liters of oxygen. The baby required 4 liters of oxygen after birth, which was weaned down over a week. The COVID-19 test for the baby was negative. Both mother and baby survived this experience without any further complications while in the hospital and were where discharged home.

## Discussion

Several physiological changes occur in the thoracic cavity during pregnancy due to the space the uterus occupies and also due to hormonal effects. As the unborn baby develops there is an increase in metabolic demand from the fetal placenta and a decrease in maternal functional residual capacity (FRC). The impaired maternal immune system predisposes pregnant patients to viral infections. In expectant patients with COVID-19, there is a decreased lung compliance which in addition to decreased FRC, increases the mother’s propensity to hypoxia. At the moment the mother registers hypoxemia either by saturation monitoring or arterial blood gases (ABG) the unborn baby would be in distress. ARDS as a complication in pregnancy has a high perinatal mortality. COVID-19 infection has direct consequences to the unborn baby by directly affecting the mother’s cardio-respiratory system. In essence, anything that causes hypoxemia to the mother will have a deleterious effect baby. There is a need for careful monitoring of these patients. continuous pulse oximetry, telemetry, and continuous fetal monitoring. The mother had progressive hypoxemia despite remdesivir and continued with increased oxygen demand. The presence of the gravid uterus and the unborn baby makes ventilation a challenge. Delivery is indicated in severe hypoxia (PaO2/FiO2 <100 for >three hours), hemodynamic instability (median artery pressure (MAP) <65 mmHg or the need of inotropic/vasoconstrictor drugs), or a life-threatening worsening of maternal conditions [3].

In ARDS using mechanical ventilation with a high PEEP, low tidal volume and allowing low plateau pressure with permissive hypercapnia, paralytics use for synchrony with the ventilator along with proning remain the cornerstones of therapy. Prone ventilation has been used during pregnancy and reported in the case report series [11]. Proning provides an augmented ventilation-perfusion ratio while decreasing the compression of the posterior and medial lung, causing a decrease in hypoxic vasoconstriction, an improvement in cardiac output, and recruitment of more alveolar units. There is a lack of randomized control trials, but the complications of standard ARDS patients included barotrauma, bleeding, transient hypotension, and transient hypoxia which could all be detrimental to the baby [8]. The need for delivery should be coordinated with a neonatologist and OB-GYN. In our experience, prompt delivery is preferable over an emergency delivery in the ICU or prior to impending cardiac arrest when the maternal status worsens. The overall experience of most intensivists in treating pregnant patients with ARDS is limited. This is a novel physiological and clinical presentation of the gravid abdomen in our ICUs. Keep in mind the goals are the same, oxygenation, maintained perfusion with cardiac output. The side effects of our treatment to the unborn baby must be considered to ensure the survival of both patients under the threat of hypoxemia.

A comparison is being added to this discussion as non-COVID-19-related ARDS management remains similar if the fetus is at a viable gestation and is at a risk due to intractable maternal hypoxia, there may be benefit to the fetus in delivery determined by standard obstetrical principles. ARDS is an uncommon condition and medical therapies such as nitric oxide and corticosteroids play a complementary role. Also, good communication between critical care, maternal-fetal medicine and obstetrics with a fundamental understanding of mechanical ventilation is essential. A decision should be based on overall risk balance to both mother and fetus for even non-COVID-related ARDS in pregnancy-related conditions such as preeclampsia, amniotic fluids embolism and viral pneumonitis [12].

## Conclusions

As we continue seeing new variants and infections of younger patients, it is important to train our ICU nurses on the importance of monitoring hemodynamics and fetal variability. Correlation between the mother’s distress and the potential hypoxemia of the unborn baby should guide us to prompt delivery. Transient hypotension and variable cardiac outputs could have long-term consequences to the unborn baby. Most ICUs in America don’t have a Maternal-Fetal Specialist, especially in the middle of a world pandemic. It is important to coordinate efforts from the Critical Care team and the Obstetrics Department and remember that all therapies are for the benefit of two. The moment either life is compromised, a lifesaving intervention to preserve both lives such as a controlled delivery should be considered. 

## References

[1] Federici L, Picone O, Dreyfuss D, Sibiude J (2020). Successful continuation of pregnancy in a patient with COVID-19-related ARDS. BMJ Case Rep.

[2] Hou L, Li M, Guo K, Wang W, Li B, Li J, Yuan Y (2021). First successful treatment of a COVID-19 pregnant woman with severe ARDS by combining early mechanical ventilation and ECMO. Heart Lung.

[3] Barile L, Cerrano M, Locatelli A, Puppo A, Signorile AF, Barzaghi N (2020). Prone ventilation in a 27 week pregnant woman with COVID-19 severe ards. Signa Vitae.

[4] Rose CH, Wyatt MA, Narang K, Lorenz KE, Szymanski LM, Vaught AJ (2021). Timing of delivery with coronavirus disease 2019 pneumonia requiring intensive care unit admission. Am J Obstet Gynecol MFM.

[5] Adhikari EH, SoRelle JA, McIntire DD, Spong CY (2022). Increasing severity of COVID-19 in pregnancy with Delta (B.1.617.2) variant surge. Am J Obstet Gynecol.

[6] Larson SB, Watson SN, Eberlein M, Simmons JS, Doerschug KC, Leslie KK (2021). Survival of pregnant coronavirus patient on extracorporeal membrane oxygenation. Ann Thorac Surg.

[7] Safaee Fakhr B, Wiegand SB, Pinciroli R (2020). High concentrations of nitric oxide inhalation therapy in pregnant patients with severe coronavirus disease 2019 (COVID-19). Obstet Gynecol.

[8] Epelboin S, Labrosse J, De Mouzon J (2021). Obstetrical outcomes and maternal morbidities associated with COVID-19 in pregnant women in France: a national retrospective cohort study. PLoS Med.

[9] Kucirka LM, Norton A, Sheffield JS (2020). Severity of COVID-19 in pregnancy: a review of current evidence. Am J Reprod Immunol.

[10] Chong J, Ahmed S, Hill K (2020). Acute respiratory distress syndrome in a pregnant patient with COVID-19 improved after delivery: a case report and brief review. Respir Med Case Rep.

[11] Cavalcante FM, Fernandes CD, Rocha LD, Galindo-Neto NM, Caetano JÁ, Barros LM (2021). Use of the prone position in pregnant women with COVID-19 or other health conditions. Rev Lat Am Enfermagem.

[12] Lapinsky SE (2015). Acute respiratory failure in pregnancy. Obstet Med.

